# Global research trends of lean metabolic dysfunction-associated steatotic liver disease (lean MASLD) from 2005 to 2024: Bibliometric and visualization analysis

**DOI:** 10.1097/MD.0000000000049183

**Published:** 2026-06-05

**Authors:** Qi Yu, Mei Liu, Liyuan He, Feng Hua

**Affiliations:** a Department of Gastroenterology, Wuxi Xinwu District Xinrui Hospital, Wuxi, Jiangsu Province, China; b Department of Gastroenterology, Wuxi Xinwu District Geriatric Hospital, Wuxi, Jiangsu Province, China.

**Keywords:** bibliometric analysis, CiteSpace, lean MASLD, visualization, VOSviewer

## Abstract

**Background:**

To analyze the research status, hotspots, and frontiers of lean metabolic dysfunction-associated steatotic liver disease (lean MASLD) in the past 20 years using bibliometrics, providing references for further related research.

**Methods:**

Literature on lean MASLD was retrieved from the Web of Science Core Collection database. Visual analyses of publication trends, author distribution, research institutions, journal distribution, cited documents, and keywords, were performed using VOSviewer, the “Bibliographic” package in R, and CiteSpace software.

**Results:**

A total of 2008 documents were included. The publication output on lean MASLD increased annually and peaked in 2023. This literature originated from 82 countries/regions, with the United States contributing the most publications. Harvard University was the institution with the highest publication count, *PLOS ONE* was the top journal in this field, and Prof Wong, Vincent Wai-Sun from The Chinese University of Hong Kong was the most productive author. The research on lean MASLD at that time primarily focused on etiology and treatment (e.g., hepatic steatosis, insulin resistance, metabolic syndrome, oxidative stress, inflammation, abdominal obesity, sarcopenia, *patatin-like phospholipase domain-containing 3* gene, and gut microbiota), as well as epidemiology and diagnostic methods.

**Conclusion:**

This study systematically depicted the 2-decade developmental trajectory of lean MASLD using bibliometric methods for the first time, and provides academic references for clinicians and scholars to grasp the field’s hotspots, frontiers, and evolutionary trends.

## 1. Introduction

Metabolic dysfunction-associated steatotic liver disease (MASLD) is defined by confirmed hepatic steatosis (≥5% hepatocytes) plus the presence of 1 of 3 criteria: overweight/obesity, type 2 diabetes, or metabolic dysregulation (≥2 metabolic syndrome components). This positive diagnostic framework replaces the exclusionary nonalcoholic fatty liver disease (NAFLD) paradigm, emphasizing metabolic dysfunction as the core pathogenesis and allowing for comorbidity with other liver diseases.^[1]^ Without timely intervention, it can progress to steatohepatitis, cirrhosis, and other metabolic complications.

NAFLD, metabolic dysfunction-associated fatty liver disease (MAFLD), and MASLD represent successive nomenclature revisions for the same disease entity. Originally defined as NAFLD based on exclusionary criteria, the terminology was subsequently updated to MAFLD to emphasize the central role of metabolic dysfunction. For international standardization, to more accurately reflect the nature of the disease and guide clinical diagnosis and management, an international expert consensus in 2024 further revised MAFLD to MASLD.^[2]^ These changes reflect a mechanistic shift from exclusion-based diagnosis to a pathogenesis-oriented definition, highlighting metabolic disturbance as the core driver of the disease.

Previous studies have suggested that MASLD is closely associated with populations, and a subset exhibits normal or even subnormal body mass index (BMI) levels,^[3–5]^ which has attracted significant attention from clinicians.

It is estimated that 7% to 20% of MASLD patients are lean. In Asian adults, lean MASLD and nonobese MASLD refer to patients with MASLD with BMI < 23 kg/m^2^ and <25 kg/m^2^, respectively. In China, these thresholds are defined as BMI < 24 kg/m^2^ and <28 kg/m^2^ for lean and nonobese MASLD, respectively.^[6]^ Ethnic-specific BMI thresholds reflect differences in body fat distribution and metabolic risk across ethnic groups.

Lean MASLD patients may share a similar pathogenesis of nonalcoholic steatohepatitis (NASH) with non-lean MASLD patients, but they differ in disease progression probability, associated comorbidities, and diagnostic/management approaches.^[7,8]^ Their etiological mechanisms, pathological manifestations, and therapeutic strategies still require in-depth investigation.

In recent years, global research on lean MASLD has expanded rapidly, with a steady surge in clinical and basic publications. Despite this growth, systematic analyses of research hotspots, evolutionary trends, and academic collaboration networks in this field are still lacking. As a robust scientific tool, bibliometrics provides critical guidance for subsequent research.^[9]^

Notably, no dedicated bibliometric analysis of lean MASLD has been reported to date. Accordingly, this study adopts bibliometric methods to systematically summarize the current status and developmental trends of lean MASLD research, aiming to provide an objective evidence base for future research directions and evidence-based clinical decision-making in this field.

## 2. Materials and methods

### 2.1. Inclusion and exclusion criteria

Inclusion criteria were as follows:

1. Literature on MAFLD published in the Web of Science Core Collection (WoSCC).
2. Study types limited to review articles and articles, with language restricted to English.
3. Publication date range: January 1, 2005, to December 31, 2024.

Exclusion criteria were as follows:

1. Literature irrelevant to lean or nonobese MASLD.
2. Duplicate studies.

### 2.2. Literature retrieval

Web of Science was chosen as the main database for this study because it encompasses over 12,000 academic journals and is frequently used by researchers. Compared with other databases such as Scopus, Medline, and PubMed, Web of Science provides the most comprehensive and reliable bibliometric analysis.^[10]^ The relevant literature on lean or nonobese NAFLD in the WoSCC was retrieved. This study did not involve animal or human experiments, so an ethical review was not required. The publication date ranged from January 1, 2005, to December 31, 2024. The retrieval strategy was as follows: TS=(“lean” OR “normal body mass index” OR “normal BMI” OR “non-obese” OR “nonobese” OR “nonobesity” OR “non obesity” OR “normal weight” OR “thin” OR “thinness” OR “underweight” OR “nonoverweight” OR “non overweight”) AND TS=(“NAFLD” OR “non-alcoholic fatty liver” OR “nonalcoholic fatty liver” OR “nonalcoholic steatohepatitis” OR “MAFLD” OR “metabolic dysfunction-associated fatty liver disease” OR “metabolic dysfunction associated fatty liver disease” OR “MASLD” OR “metabolic dysfunction-associated steatotic liver disease” OR “metabolic dysfunction associated steatotic liver disease”).

The retrieval link is as follows: https://webofscience.clarivate.cn/wos/woscc/summary/18d2a48a-22bf-4f52-83b3-9379de40aac6-0162d42bac/relevance/1.

### 2.3. Literature screening and data extraction

Duplicate literature was removed using CiteSpace software. Two researchers read and screened the titles and abstracts of the retrieved literature according to the inclusion and exclusion criteria, and any disagreements were discussed with a third researcher. Relevant data from the included literature were extracted, including authors, countries, publication years, research institutions, keywords, citation status, and publication journals.

### 2.4. Bibliometric analysis methods

VOSviewer, designed by van Eck and Waltman, is software for visualizing collaborative networks and research themes in academic fields.^[11]^ In this study, VOSviewer (version 1.6.19), developed by the Centre for Science and Technology Studies at Leiden University, the Netherlands, was used to analyze author and institutional collaborations in the field of lean MASLD research.

CiteSpace, a citation metrics software developed by Professor Chaomei Chen and collaborators, facilitates visualization and analysis of complex citation networks.^[12]^ Using CiteSpace (version 6.2.R4 Advanced) developed by Dr Chaomei Chen at Drexel University, we conducted keyword visualization, clustering, timeline mapping, and burst term analysis.

The bibliometrix package in R is a powerful and flexible tool for in-depth bibliometric data analysis.^[13]^ Employing Bibliometrix (R-studio, R-tool, version 4.3.2) developed by Massimo Aria and Corrado Cuccurullo at the University of Tor Vergata, Rome, Italy, we performed analyses of regional/institutional/journal distributions and keywords via the online bibliometric analysis platform (http://bibliometric.com/).

Annual publication trends were analyzed using Microsoft Excel (2021 edition), developed by Microsoft.

## 3. Results

### 3.1. Analysis of research status

#### 3.1.1. Annual publication trends of included literature

**Figure 1.**
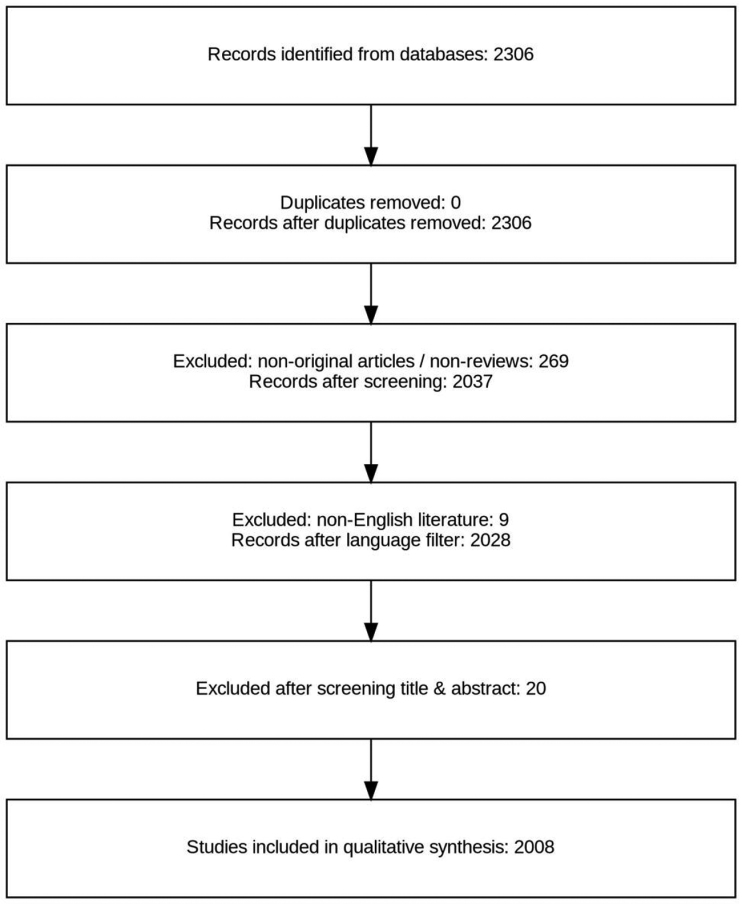
Flowchart of study.

A total of 2306 literature items were initially retrieved from the WoSCC database. After deduplication and screening according to the exclusion criteria, 2008 literature items related to lean or nonobese NAFLD were finally included. The PRISMA flowchart is shown in Figure 1. A total of 1811 articles and 197 reviews were included, as shown in Table 1.

**Table 1:** Inclusion of literature distribution.

Document	Number	Times cited	H-index
Article	1811	76,760	130
Review	197	20,669	68

**Figure 2.**
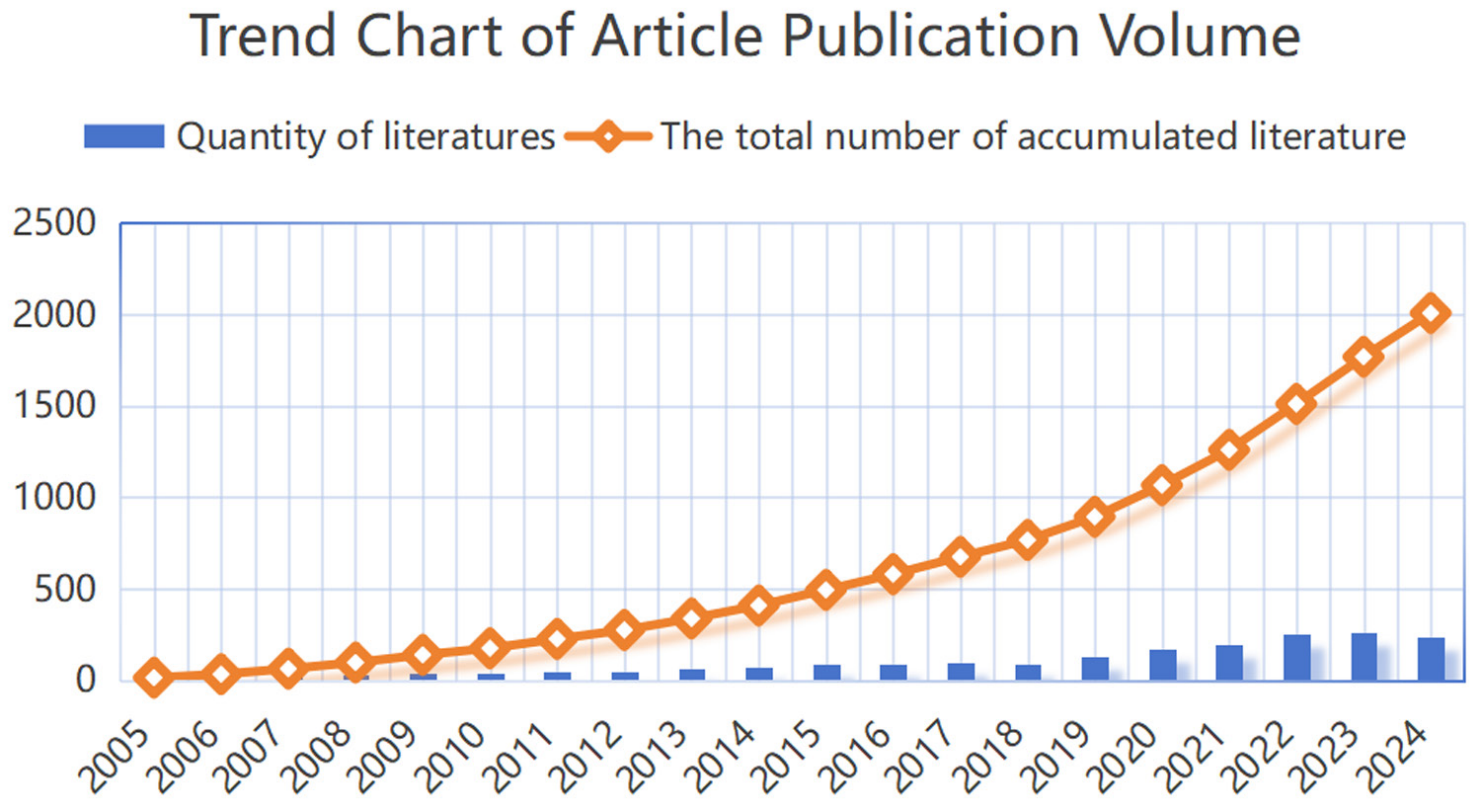
Annual publication trend chart of lean MASLD. MASLD = metabolic dysfunction-associated steatotic liver disease.

The number of relevant studies was small and increased slowly from 2005 to 2010, indicating an initial stage of research. Publication volume began to increase steadily from 2011 to 2017; a marked growth was observed from 2018 to 2024, with the number of literature items significantly rising. The publication output peaked in 2023 (258 articles), followed by a brief decline and stabilization (see Fig. 2).

#### 3.1.2. National/regional and institutional distribution of published literature

**Figure 3.**
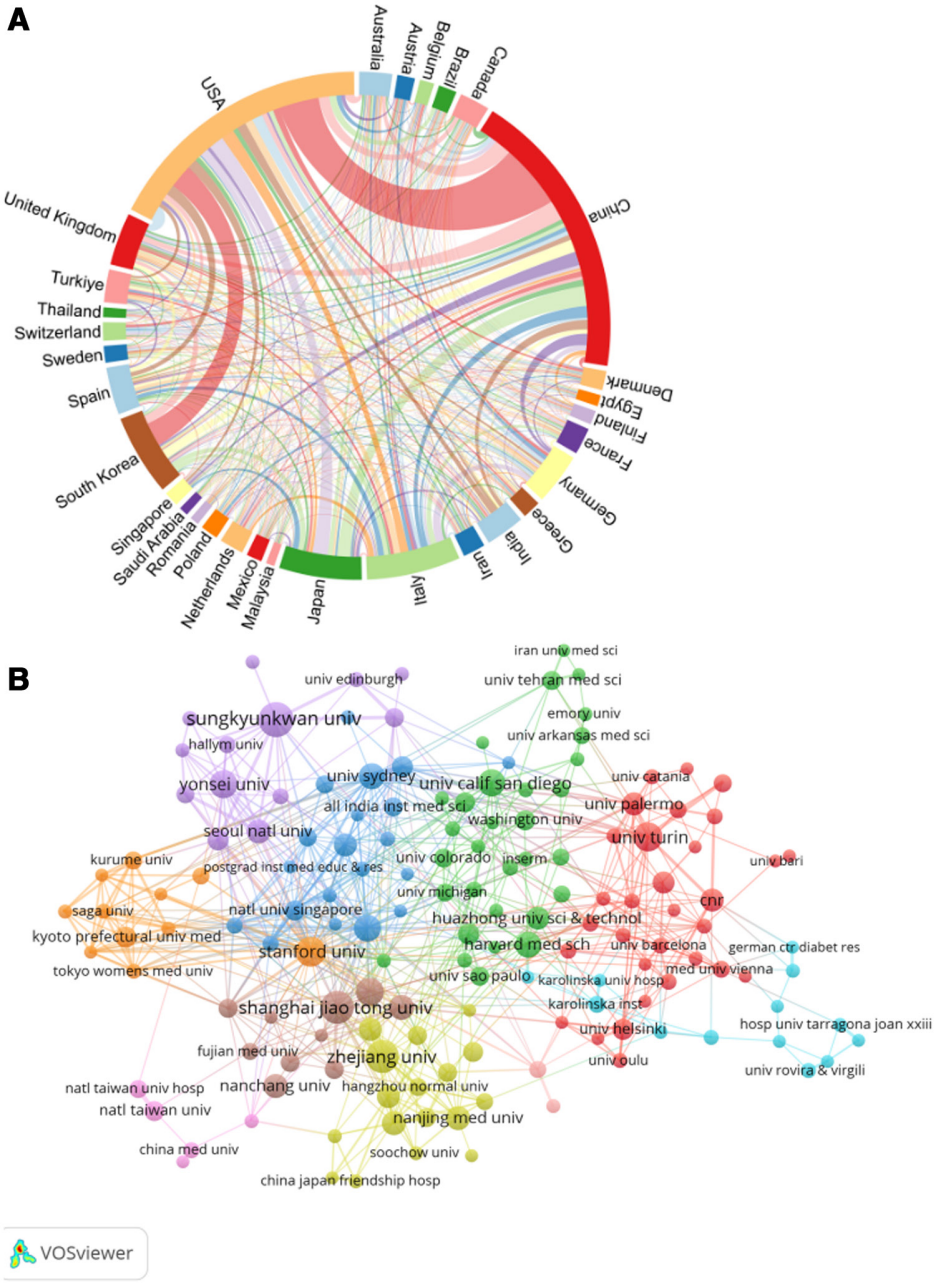
Cooperative network diagram of countries/institutions for lean MASLD research. Here, (A) represents the distribution map of research countries and (B) represents the distribution map of research institutions. MASLD = metabolic dysfunction-associated steatotic liver disease.

The online bibliometric analysis platform (http://bibliometric.com/) was used to count the number of articles published in 82 countries/regions, aiming to identify those playing a leading role in lean MASLD research (Fig. 3A). Different colors represent countries, the arc length indicates publication volume, and the connecting lines in the middle signify collaborative relationships – the thicker the line, the closer the collaboration.

The United States ranked first with the highest global publication output (502 articles), followed by China (482 articles), and Italy in third place. The most critical research collaboration existed between China and the United States. Close ties were also maintained between the United States and South Korea, followed by collaboration between the United States and Japan.

In bibliometrics, betweenness centrality is an important indicator for measuring the perceived weight of network nodes. A higher betweenness centrality indicates that the node plays a more critical bridging role in the flow and transmission of knowledge within the network. According to the analysis results of VOSviewer, the number of publications and centrality are the core factors considered when determining the 5 most influential countries/regions, and the specific rankings are shown in Table 2. The United States ranked first with a total of 502 publications, and its centrality score of 0.39 also firmly ranked first.

**Table 2:** National publication volume of lean MASLD.

Rank	Country	Number of publications	Country	Betweenness centrality
1	United States	502	United States	0.39
2	China	482	Spain	0.14
3	Italy	173	England	0.12
4	Japan	153	Australia	0.11
5	South Korea	151	Saudi Arabia	0.11

MASLD = metabolic dysfunction-associated steatotic liver disease.

A total of 2790 institutions participated in related research. The top 5 institutions ranked by publication quantity and betweenness centrality are shown in Table 3. Harvard University ranked first with 43 publications, followed by Shanghai Jiao Tong University (42 publications). The French National Institute of Health and Medical Research had the highest network centrality, with a centrality score of 0.29, followed by The Chinese University of Hong Kong (0.23).

**Table 3:** National publication volume of lean MASLD.

Rank	Country	Number of publications	Institution	Betweenness centrality
1	Harvard University	43	The French National Institute of Health and Medical Research (INSERM)	0.29
2	Shanghai Jiao Tong University	42	The Chinese University of Hong Kong	0.23
3	University of California System	41	Stanford University	0.21
4	Harvard Medical School Affiliated Hospital	37	Harvard Medical School	0.14
5	Sungkyunkwan University (SKKU)	34	China National Center for Biomedical Research	0.12

MASLD = metabolic dysfunction-associated steatotic liver disease.

VOSviewer software was used to analyze the institutional collaboration network (Fig. 3B). Each node represents a research institution, the size of the node indicates the number of publications of the institution, the connecting lines between nodes signify collaboration, and colors are used to distinguish different collaboration clusters.^[14]^ The research field of lean MASLD can be divided into 9 closely related clusters.

#### 3.1.3. Journal distribution

The online bibliometric analysis platform (http://bibliometric.com/) was used to analyze journal influence. Table 4 lists the top 5 journals in terms of publication quantity in the field of lean MASLD. The top 3 are *PLOS ONE* (48 articles, impact factor [IF] 2.7), *Hepatology* (47 articles, IF 13.0), and *Scientific Reports* (46 articles, IF 3.2).

**Table 4:** Top 5 journals with the highest publication output on lean MASLD.

Rank	Journal	Number of publications	IF (2024)	JCR
1	PLoS One	48	2.9	Q2
2	Hepatology	47	13	Q1
3	Scientific Reports	46	3.2	Q2
4	Nutrients	42	4.8	Q1
5	Liver International	41	6.1	Q1

IF = impact factor, JCR = Journal Citation Reports, MASLD = metabolic dysfunction-associated steatotic liver disease.

Table 5 shows the top 10 journals by citation frequency. The top 3 are *Hepatology* (IF 13.0), *J Hepatol* (IF 26.8), and *Gastroenterology* (IF 26.3). More than half of these journals are from the United States. The high IF values indicate the quality of literature in the field of lean MASLD.

**Table 5:** Top 5 journals with the highest citation counts for lean MASLD.

Rank	Journal	Citation counts	IF (2024)	JCR
1	Hepatology	6910	13	Q1
2	J Hepatol	3848	26.8	Q1
3	Gastroenterology	2777	26.3	Q1
4	PLoS One	1595	2.9	Q2
5	J Clin Endocr Metab	1535	5	Q1

IF = impact factor, JCR = Journal Citation Reports, MASLD = metabolic dysfunction-associated steatotic liver disease.

#### 3.1.4. Author distribution of published literature

**Figure 4.**
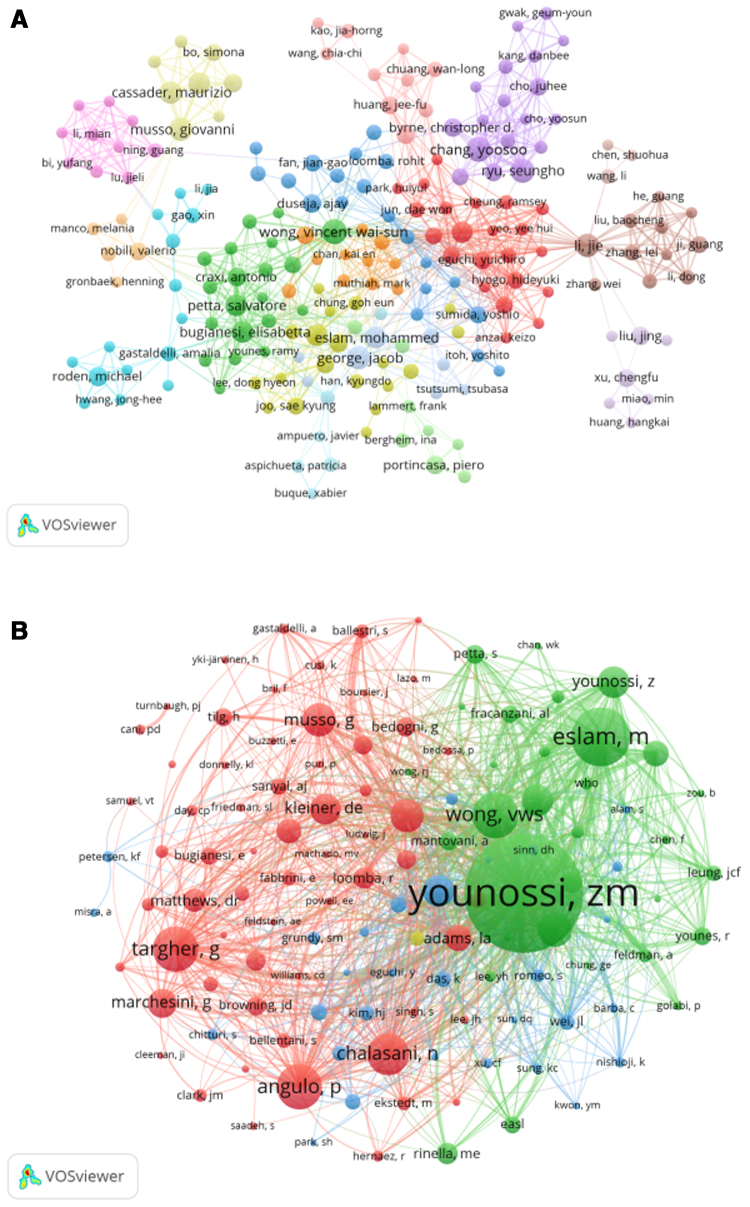
Co-occurrence network map of authors in lean MASLD literature. Here, (A) shows the co-occurrence network of research authors and (B) shows the co-occurrence network of co-cited authors. MASLD = metabolic dysfunction-associated steatotic liver disease.

VOSviewer analysis showed that the top 10 most productive authors in lean MASLD research are Wong, Vincent Wai-Sun (17 articles), Chang, Yoosoo (16 articles), Aguilar, Carmen (16 articles), Auguet, Teresa (16 articles), Richart, Cristobal (16 articles), and Ryu, Seungho (16 articles), as shown in Figure 4A. Productive and highly cited authors exhibit concentrated network nodes with close connections, indicating tight collaboration among scholars. Specific information on authors is listed in Table 6.

**Table 6:** Top 5 authors with the highest publication and citation counts on lean MASLD.

Rank	Author	Counts	Institution	Co-cited author	Citations	Institution
1	Wong, Vincent Wai-Sun	17	The Chinese University of Hong Kong	Younossi, Zobair M.	1030	Inova Fairfax Hosp
2	Chang, Yoosoo	16	Sungkyunkwan University (SKKU)	Eslam, Mohammed	511	The University of Sydney
3	Aguilar, Carmen	16	Universitat Rovira i Virgili	Wong, Vincent Wai-Sun	403	The Chinese University of Hong Kong
4	Auguet, Teresa	16	Univ Rovira i Virgili URV	Targher, Giovanni	396	IRCCS Sacro Cuore Don Calabria Hosp
5	Richart, Cristobal	16	Joan XXIII Univ Hosp Tarragona	Angulo, P	392	Mayo Clin

MASLD = metabolic dysfunction-associated steatotic liver disease.

Two authors are defined as co-cited authors if their works are cited together by a later publication. A higher co-citation strength suggests that these authors share closer research topics, theoretical foundations, or methodological perspectives, and it is widely used to identify research communities and knowledge clusters. The top 3 most co-cited authors are Younossi, Zobair M (1030 citations), Eslam, Mohammed (511 citations), and Wong, Vincent Wai-Sun (403 citations), as shown in Figure 4B.

### 3.2. Analysis of research hotspots and frontiers

#### 3.2.1. Analysis of literature citation frequency

Highly cited articles are important indicators of influence in a research field. The top 10 most cited literature^[15–24]^ are shown in Table 7. The most cited work is by Younossi et al,^[15]^ published in *Nature Reviews Gastroenterology & Hepatology* in 2018, with 3790 citations. Notably, this author also ranks first in co-citation. The review focuses on the global epidemiology, pathogenesis, and clinical outcomes of lean MASLD, as well as challenges such as patient identification, risk factors, and insufficient research data.

**Table 7:** Top 10 articles with the highest citation counts.

Rank	Title	Author	Journal	Year	Citations	Type	DOI	IF/JCR
1	Global burden of NAFLD and NASH: trends, predictions, risk factors and prevention	Younossi, Z	Nature Reviews Gastroenterology & Hepatology	2018	3790	Review	10.1038/nrgastro.2017.109	45.9/Q1
2	New trends on obesity and NAFLD in Asia	Fan, JG	Journal of Hepatology	2017	814	Review	10.1016/j.jhep.2017.06.003	26.8/Q1
3	Adaptation of hepatic mitochondrial function in humans with non-alcoholic fatty liver is lost in steatohepatitis	Koliaki, C	Cell Metabolism	2015	754	Article	10.1016/j.cmet.2015.04.004	27.7/Q1
4	Prevalence, incidence, and outcome of nonalcoholic fatty liver disease in Asia, 1999–2019: a systematic review and meta-analysis	Li, J	Lancet Gastroenterology & Hepatology	2019	719	Review	10.1016/S2468-1253(19)30039-1	30.9/Q1
5	Linking gut microbiota and inflammation to obesity and insulin resistance	Saad, MJA	Physiology	2016	597	Article	10.1152/physiol.00041.2015	5.3/Q1
6	Global prevalence, incidence, and outcomes of non-obese or lean non-alcoholic fatty liver disease: a systematic review and meta-analysis	Ye, Q	Lancet Gastroenterology & Hepatology	2020	587	Review	10.1016/S2468-1253(20)30077-7	30.9/Q1
7	Liver PPARα is crucial for whole-body fatty acid homeostasis and is protective against NAFLD	Montagner, A	Gut	2016	541	Article	10.1136/gutjnl-2015-310798	23.1/Q1
8	Insulin resistance drives hepatic de novo lipogenesis in nonalcoholic fatty liver disease	Smith, GI	Journal of Clinical Investigation	2020	495	Article	10.1172/JCI134165	13.3/Q1
9	High prevalence of nonalcoholic fatty liver disease in patients with type 2 diabetes mellitus and normal plasma aminotransferase levels	Portillo-Sanchez, P	Journal of Clinical Endocrinology & Metabolism	2015	394	Article	10.1210/jc.2015-1966	5.0/Q1
10	Determinants of fibrosis progression and regression in NASH	Schuppan, D	Journal of Hepatology	2018	369	Review	10.1016/j.jhep.2017.11.012	26.8/Q1

IF = impact factor, JCR = Journal Citation Reports, NAFLD = nonalcoholic fatty liver disease, NASH = nonalcoholic steatohepatitis, PPARα = peroxisome proliferator-activated receptor alpha.

Fan et al^[16]^ published a review in the *Journal of Hepatology* in 2017, indicating that 8% to 19% of Asians with a BMI < 25 kg/m^2^ have MASLD, known as “lean” or “non-obese” MASLD. For lean MASLD, the pathological processes of steatohepatitis and fibrosis are recognized. Central obesity, insulin resistance (IR), and weight gain are major risk factors, while genetic susceptibility (e.g., *patatin-like phospholipase domain-containing 3* [*PNPLA3*] gene polymorphism) plays a more critical role in MASLD development among nonobese populations. Lifestyle modification remains the cornerstone of treatment for obesity and MASLD, and future studies should determine optimal management strategies for obesity and MASLD in Asian populations.

#### 3.2.2. Research trend analysis based on keyword analysis

**Figure 5.**
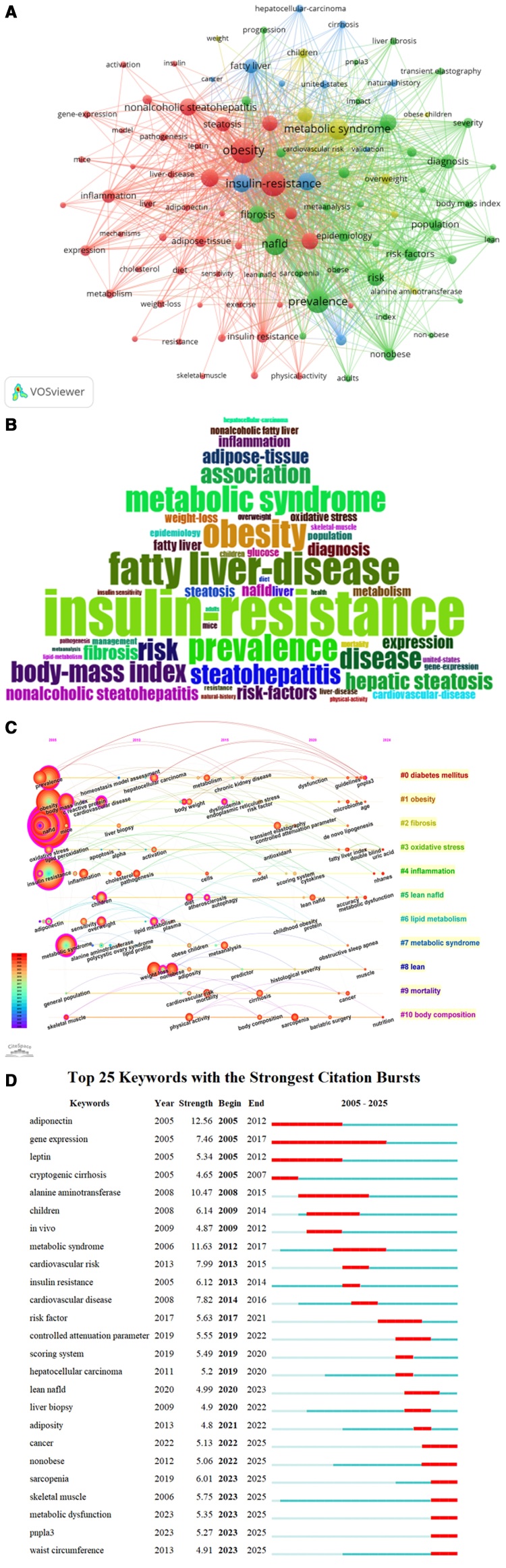
Keyword analysis of lean MASLD. (A) Shows the visual analysis of keywords. (B) shows the keyword cloud. (C) depicts the timeline map of keywords. (D) shows the keyword burst analysis. MASLD = metabolic dysfunction-associated steatotic liver disease.

Keyword analysis is a vital component of bibliometrics, facilitating the understanding of research frontiers and hotspots in a field. Table 8 presents the top 20 keywords by occurrence frequency, with “nonalcoholic fatty liver disease” (n = 1554) ranking highest, followed by “hepatic steatosis” (n = 794) and “insulin resistance” (n = 784). Visual analysis of keywords using VOSviewer generated 4 clustering groups (Fig. 5A).

**Table 8:** High-frequency keywords in the field of lean MASLD.

Rank	Keywords	Frequencies	Rank	Keywords	Frequencies
1	NAFLD	1554	11	inflammation	164
2	hepatic steatosis	794	12	nonobese	163
3	insulin resistance	784	13	cardiovascular disease	135
4	obesity	718	14	children	135
5	prevalence	519	15	pathogenesis	102
6	metabolic syndrome	417	16	overweight	96
7	liver fibrosis	350	17	oxidative stress	90
8	body mass index	259	18	mortality	75
9	diabetes mellitus	247	19	gene expression	73
10	weight loss	232	20	gut microbiota	68

MASLD = metabolic dysfunction-associated steatotic liver disease, NAFLD = nonalcoholic fatty liver disease.

The keyword word cloud generated by http://bibliometric.com/ is shown in Figure 5B. Word clouds are a visualization method for displaying keyword frequencies in text data, where the visual size of keywords is typically proportional to their occurrence frequency. “Insulin resistance,” “fatty liver,” and “metabolic syndrome” emerge as prominent research themes in this field.

Keywords were visualized using CiteSpace to generate a keyword timeline map (Fig. 5C). The map uses circular nodes to represent keywords, with node size corresponding to keyword frequency. The spectrum from dark to light indicates the chronological order, and lines between nodes denote keyword associations. This timeline map shows the most frequent keywords in each cluster during specific periods, indicating that keywords in the lean MASLD research field can be divided into 11 clusters: #0 (diabetes mellitus), #1 (obesity), #2 (fibrosis), #3 (oxidative stress), #4 (inflammation), #5 (lean NAFLD), #6 (lipid metabolism), #7 (metabolic syndrome), #8 (lean), #9 (mortality), and #10 (body composition).

Keyword burst analysis is another approach to identifying research hotspots and frontiers. In the figure, “Begin” denotes the start time of keyword bursts, “End” the end time, and “Strength” the intensity of keyword bursts, which is directly proportional to the keyword’s influence.^[12]^

The top 10 burst keywords are shown in Figure 5D. Analysis revealed that 4 keywords emerged in 2005, with research hotspots at that time focusing on pathogenesis mechanisms (adiponectin, gene expression, leptin, cryptogenic cirrhosis). Since 2008, attention has turned to childhood incidence and laboratory indicators. Metabolic syndrome, cardiovascular risk, and IR became hotspots in 2012. From 2019 onwards, discussions centered on diagnostic criteria such as controlled attenuation parameter, scoring systems, and liver biopsy. Since 2022, research hotspots have focused on sarcopenia (skeletal muscle metabolic dysfunction), the *PNPLA3* gene, and waist circumference (abdominal obesity).

### 3.3. Multivariate relationship network analysis

**Figure 6.**
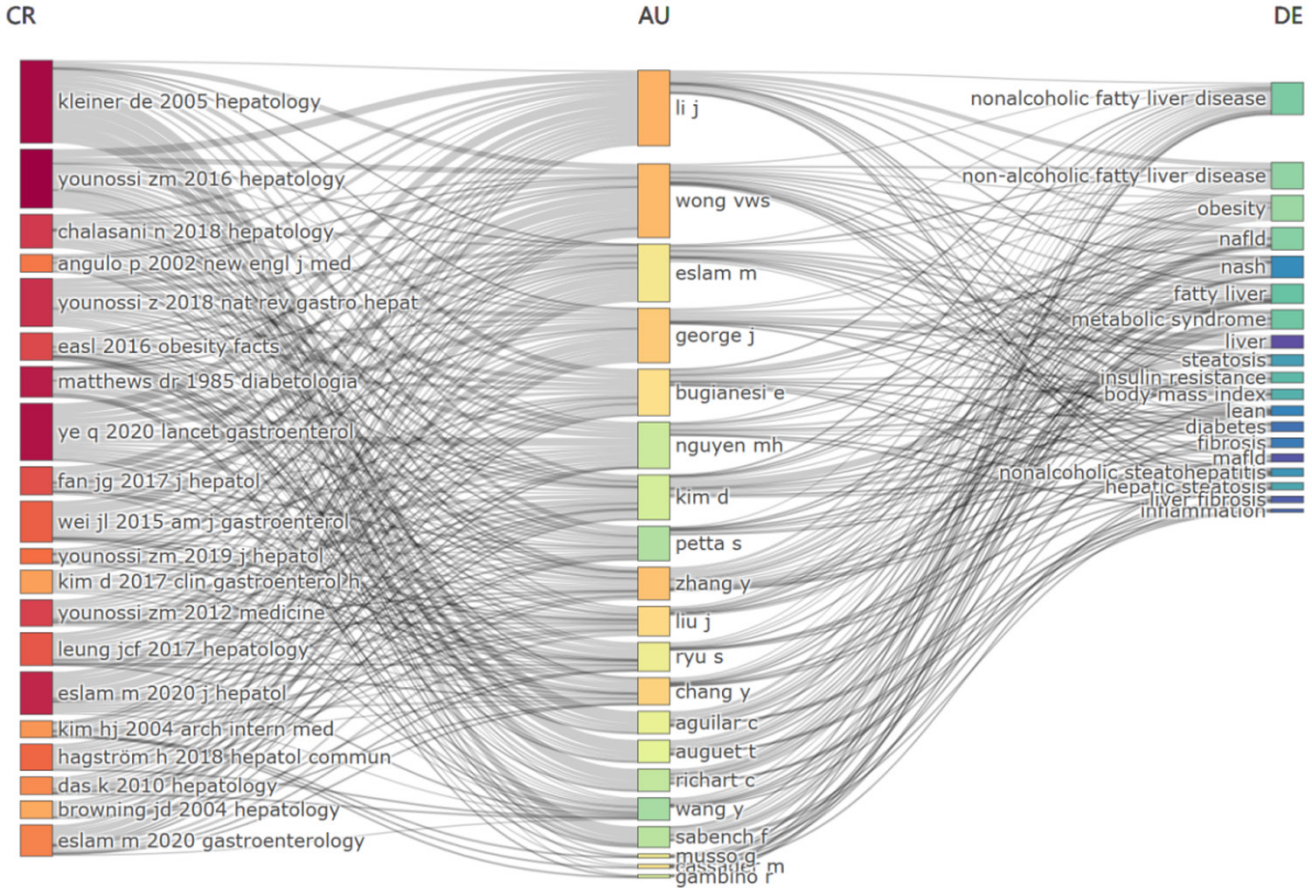
Multivariate relationship network diagram of lean MASLD. MASLD = metabolic dysfunction-associated steatotic liver disease.

Multivariate relationship network analysis was performed using the online bibliometric platform (http://bibliometric.com/). Three variables – references, authors, and keywords – were analyzed via a 3-line diagram, where the strength of associations is indicated by the width of connecting lines, and the height of rectangles represents publication quantity. As shown in Figure 6, Li J, Wong VWS, and Eslam M rank among the top 3 based on line width, indicating their high frequency of occurrence. These authors also cited highly referenced literature. Li et al^[18]^ extensively cited several references listed on the left in their articles, with the most frequently cited being Younossi ZM’s 2016 publication. Meanwhile, Wong VWS’s research involves high-frequency keywords such as “non-alcoholic fatty liver disease,” “lean phenotype,” “obesity,” “metabolic syndrome,” “insulin resistance,” and “fibrosis.”

## 4. Discussion

This study conducted a visual analysis of 2008 publications in the field of lean MASLD retrieved from Web of Science between 2005 and 2024, revealing information on spatiotemporal distribution, key authors, core keywords, and critical citations to explore developmental trends and research hotspots in this field.

### 4.1. Research status

From 2005 to 2024, the overall publication output on lean MASLD showed continuous growth, with a sustained explosive increase since 2018, indicating that this field has attracted global researchers’ attention.

A total of 1811 articles and 197 reviews were included. The articles were cited 76,760 times with an h-index of 130, while the reviews were cited 20,669 times with an h-index of 68. The included studies were dominated by original articles. Although reviews constituted a small proportion, they exhibited higher citation efficiency and academic impact, indicating an overall high-quality research foundation.

In terms of publication quantity, the United States and China lead the field of lean MASLD, with their combined output accounting for nearly half of the total. The United States has the highest publication volume and the highest betweenness centrality, reflecting its leading international influence. This may be attributed to NASH being the primary cause of the rapid increase in the incidence of cirrhosis and liver cancer, as well as the demand for liver transplantation in the United States,^[25]^ while lean MASLD shows higher prevalence in Asian regions such as China. In terms of collaboration, the United States is most closely connected with other countries, demonstrating its central role in global academic activities.

In institutional publication statistics, Harvard University is the most productive institution. Although the French National Institute of Health and Medical Research does not have the highest publication volume, its highest centrality indicates significant influence in this field. China ranks second in publication quantity, but both national and institutional centrality remain low. In future development, it is necessary to establish effective and rational cooperation mechanisms, promote cross-regional collaboration, strengthen international exchanges, and enhance global influence.

Regarding journal distribution, *PLOS ONE* publishes the most articles in this field. The top 5 journals by publication quantity have relatively low average IFs, while the top 5 by citation frequency have an average IF of 14.8, suggesting a need for more in-depth and high-quality research in this domain.

Analyses of high-productivity authors and co-cited authors help identify key figures in the lean MASLD field. Co-cited authors are typically scholars with high influence in the same field, whose research is considered crucial for field development and promotion. Among contributors to lean MASLD, Professor Vincent Wai-Sun Wong from The Chinese University of Hong Kong (17 articles) and Yoosoo Chang from Sungkyunkwan University (16 articles) have the highest publication outputs. Professor Wong focuses on the epidemiology of MASLD prevalence in global and Asian populations, as well as the noninvasive assessment of liver fibrosis, while Chang specializes in MASLD pathogenesis and its association with metabolic disease risks. Both have promoted the renaming and further research of MASLD. The top 3 most cited authors are Zobair M. Younossi, Mohammed Eslam, and Vincent Wai-Sun Wong, with Eslam from the University of Sydney and Wong being the most productive author.

### 4.2. Research hotspots

Analysis of the top 10 most cited articles shows they were published relatively late (2015–2020), indicating that while global attention to MASLD emerged early, research on lean MASLD started later. Among them, 5 review articles cover global and Asian epidemiology, prognosis of MASLD, and diagnosis/progression of liver fibrosis,^[15,16,18,20,24]^ while the other 5 original studies focus on pathogenesis, including liver peroxisome proliferator-activated receptor alpha, gut microbiota, IR, and inflammation.^[17,19,21–23]^

As keywords highly summarize literature content, keyword analysis is central to bibliometric studies for exploring potential research hotspots. This study reveals that current research on lean MASLD mainly focuses on:

- Etiology and treatment: hepatic steatosis, IR, metabolic syndrome, oxidative stress, inflammation, abdominal obesity, sarcopenia, *PNPLA3* gene, and gut microbiota.
- Epidemiology and diagnostic methods.

Background of research hotspots:

1. Studies have shown that lean MASLD predominantly affects elderly men and is often accompanied by extrahepatic diseases, including diabetes, hyperlipidemia, hypertension, metabolic syndrome, chronic kidney disease, and cardiovascular diseases.^[26,27]^ In a large cohort of nonobese Asian subjects, the severity of MASLD is associated with increased cardiovascular risk, exhibiting similar severe histological phenotypes to obese MASLD.^[28]^
2. *PNPLA3* gene influence: *PNPLA3* is one of the first genes associated with NAFLD. In lean patients with high-sucrose diet-induced *PNPLA3* p.I148M mutation, carriers face an increased risk of NASH and fibrosis ≥stage 2.^[29,30]^ This suggests *PNPLA3* may exert a greater impact on lean MASLD than on obese MASLD, warranting closer monitoring of liver progression in lean patients carrying this gene variant.
3. Gut microbiota role: Lipopolysaccharides produced by gut microbiota induce chronic subclinical inflammation and obesity, triggering IR via toll-like receptor 4 activation. Lean MASLD patients exhibit altered gut microbiota profiles. A study by Chen et al^[31]^ showed that treating mice with the apical sodium-dependent bile acid transporter inhibitor (SC-435) increased fibroblast growth factor 15, altered bile acid and microbial distribution, and improved lean steatohepatitis. Metabolic adaptation differences between lean and non-lean MASLD patients offer new therapeutic options.^[32]^
4. Microbiota dysbiosis in MASLD: Compared to healthy individuals, NAFLD patients show microbial dysbiosis, with increased abundance of Streptococcus, Escherichia, Anaeroglobus, and Bilophila, which enhance endotoxemia, produce endogenous ethanol, and promote inflammation/IR, and decreased abundance of Lactococcus, Coprococcus, *Faecalibacterium prausnitzii*, Alistipes, Bifidobacterium, and Akkermansia, reducing short-chain fatty acid production, intestinal mucosal integrity maintenance, pathogen inhibition, and anti-inflammatory effects.^[33,34]^
5. Sarcopenia as a risk factor: Sarcopenia (progressive loss of skeletal muscle mass, strength, and function) is another risk factor for MASLD in normal-weight individuals. A bidirectional interaction exists between sarcopenia and IR. Skeletal muscle plays a critical role in insulin-mediated glucose metabolism. Muscle loss impairs glucose metabolism and exacerbates IR.^[35]^ Sarcopenia reduces exercise tolerance, decreasing energy expenditure and promoting weight gain/IR.^[36]^
6. Chronic inflammation and reactive oxygen species overload from abnormal cytokines in MASLD patients damage skeletal muscle, particularly in lean/nonobese populations.^[37]^
7. Diagnostic indicators and risk stratification:
  a. Key predictive indices for MASLD:
    i. Liver fat score, visceral adiposity index, and lipid accumulation product index show high predictive value for NAFLD.^[38]^
    ii. Fibrosis-4 index and NAFLD fibrosis score have been validated for excluding liver fibrosis.^[39,40]^
    iii. Specific thresholds for lean NAFLD: A fatty liver index ≥15 serves as a critical value for screening lean NAFLD, while a fatty liver index ≤5 has a 95% negative predictive value.^[41]^
  b. Risk stratification and diagnostic approaches:
    i. All lean NAFLD patients should undergo noninvasive testing for risk stratification to identify those at the highest risk of disease progression.^[42]^
    ii. Liver biopsy is recommended as a reference standard when the cause of liver injury and/or fibrosis stage remains uncertain.^[43]^
8. Prognosis and clinical implications:
  a. Paradigm shift in prognostic understanding: Historically, lean MASLD was considered to have a better prognosis due to fewer obesity-related comorbidities. However, emerging evidence shows lean and non-lean MASLD patients face similar risks of metabolic and cardiovascular diseases.^[44]^
  b. Elevated risks in lean subgroup: Cheah et al analyzed data from more than 10.01 million individuals worldwide, focusing on lean MASLD defined by normal BMI. The results demonstrated that lean MASLD is an underrecognized high-risk phenotype, with a metabolic burden comparable to that of obese MASLD but associated with a higher risk of liver-related mortality.^[45]^ A population-based retrospective cohort study published in Clinical Gastroenterology and Hepatology in 2022 included 4834 patients with NAFLD (414 in the normal BMI group, 1189 in the overweight group, and 3231 in the obese group), with a median follow-up of approximately 19.9 years, comparing the natural history and prognosis of NAFLD patients across different BMI categories. The results showed that the normal BMI group had a significantly higher all-cause mortality (hazard ratio = 1.96, 95% confidence interval: 1.52–2.51), which remained significant after adjustment for confounders.^[46]^ This underscores the need for enhanced early identification and scientific management of lean MASLD populations.

The above summary of current research hotspots is presented as relevant background knowledge to facilitate the interpretation of bibliometric results rather than an in-depth expansion of literature not covered by this study.

### 4.3. Study limitations

This research has several limitations:

Database dependency: Although WoSCC is one of the most comprehensive and authoritative databases for bibliometric analysis, relying solely on its data may lead to incomplete retrieval and affect study outcomes.
Retrieval constraints: Strict limitations on search formulas, literature types, and languages may exclude some relevant articles, potentially biasing the findings.
Sample size limitation: Despite the recent growth in publications, the overall number of studies in this field remains relatively small, which may introduce slight deviations in analysis results based on existing literature.

Future studies should strive to address and mitigate these limitations.

## 5. Conclusion

In summary, this study comprehensively and objectively analyzed literature in the field of lean MASLD based on bibliometric analysis. Research on lean MASLD has shown sustained growth and is still developing rapidly. The United States and China have made prominent contributions to the field, yet China’s global academic influence needs further improvement. Current research hotspots focus on diagnosis, etiology, and treatment. There is an urgent need to strengthen cooperation and exchanges between nations and institutions – particularly among Asian countries with high incidence rates – to promote field development and benefit more patients with lean MASLD.

## Author contributions

**Conceptualization:** Mei Liu.

**Methodology:** Qi Yu, Mei Liu, Feng Hua.

**Data curation:** Qi Yu, Liyuan He.

**Project administration:** Liyuan He.

**Supervision:** Liyuan He, Feng Hua.

**Formal analysis:** Feng Hua.

**Writing – original draft:** Qi Yu, Mei Liu.

**Writing – review & editing:** Qi Yu, Mei Liu.
